# Correction: Jugel et al. Targeted Transposition of Minicircle DNA Using Single-Chain Antibody Conjugated Cyclodextrin-Modified Poly (Propylene Imine) Nanocarriers. *Cancers* 2022, *14*, 1925

**DOI:** 10.3390/cancers18030360

**Published:** 2026-01-23

**Authors:** Willi Jugel, Stefanie Tietze, Jennifer Daeg, Dietmar Appelhans, Felix Broghammer, Achim Aigner, Michael Karimov, Gabriele Schackert, Achim Temme

**Affiliations:** 1 Department of Neurosurgery, Section Experimental Neurosurgery and Tumor Immunology, University Hospital Carl Gustav Carus, TU Dresden, Fetscherstraße 74, 01307 Dresden, Germany; willi.jugel@uniklinikum-dresden.de (W.J.); stefanie.tietze@uniklinikum-dresden.de (S.T.); felix.broghammer@uniklinikum-dresden.de (F.B.); gabriele.schackert@uniklinikum-dresden.de (G.S.); 2 Leibniz Institute of Polymer Research Dresden e.V., Mailbox 120411, 01069 Dresden, Germany; jenniferdaeg@web.de (J.D.); applhans@ipfdd.de (D.A.); 3 Rudolf-Boehm-Institute for Pharmacology and Toxicology, Clinical Pharmacology, Faculty of Medicine, University of Leipzig, 04107 Leipzig, Germany; achim.aigner@medizin.uni-leipzig.de (A.A.); michael.karimov@medizin.uni-leipzig.de (M.K.); 4 German Cancer Consortium (DKTK), Partner Site Dresden, 01307 Dresden, Germany; 5 German Cancer Research Center (DKFZ), 69120 Heidelberg, Germany; 6 National Center for Tumor Diseases (NCT), 01307 Dresden, Germany

## Error in Figure

In the original publication [1], there was a mistake in Figure 2C, as published. It was noticed that an unsuitable image was inadvertently implemented for a Coomassie-stained SDS-PAGE gel demonstrating the purity of the recombinant antibody lots. The corrected Figure 2C appears below and depicts the purity of the recombinant antibody lots used for the experiments. The authors state that the scientific conclusions are unaffected. This correction was approved by the Academic Editor. The original publication has also been updated.


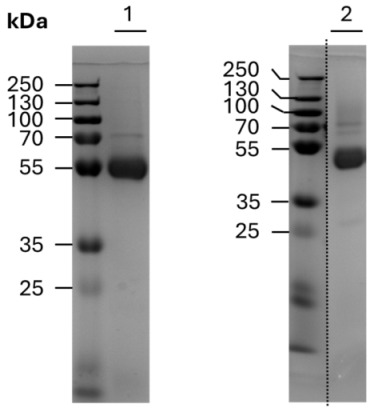


## Supplementary Materials

Uncroppped PAGE gels corresponding to corrected Figure 2C are included as Figure S5 in the Supplementary Material.
