# Supplementary material for: Targeted Transposition of Minicircle DNA Using Single-Chain Antibody Conjugated Cyclodextrin-Modified Poly (Propylene Imine) Nanocarriers
Source: Cancers (Basel). 2022 Apr 11;14(8):1925. doi: 10.3390/cancers14081925 (PMC9027598; doi:10.3390/cancers14081925)
Supplement: Supplementary file 1 [file cancers-14-01925-s001.zip › cancers-1650663-supplementary.pdf]

Supplementary Materials:

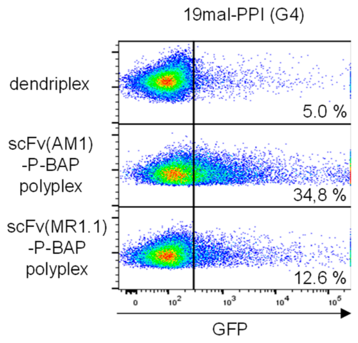

Figure S1 Representative dot plot analysis of 293T^{PSCA} cells, transfected with MC-GFP using scFv(AM1)-P-BAP-guided mal19-PPI (G4) polyplexes in comparison to PSCA-unspecific scFv(MR1.1)-P-BAP-mal19-PPI (G4) polyplexes and mal19-PPI (G4) dendriplexes.

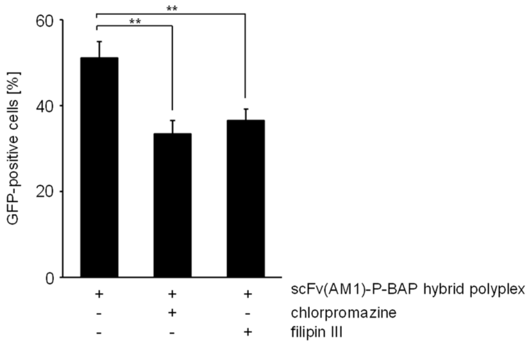

Figure S2 Transfection efficiencies of GFP expression in 293T^{PSCA} cells treated with scFv(AM1)-P-BAP-guided and scFv(MR1.1)-P-BAP-guided hybrid polyplexes containing MC that encode GFP in the presence of inhibitors of endocytosis filipin III and chlorpromazine (n = 3, mean ± SD).

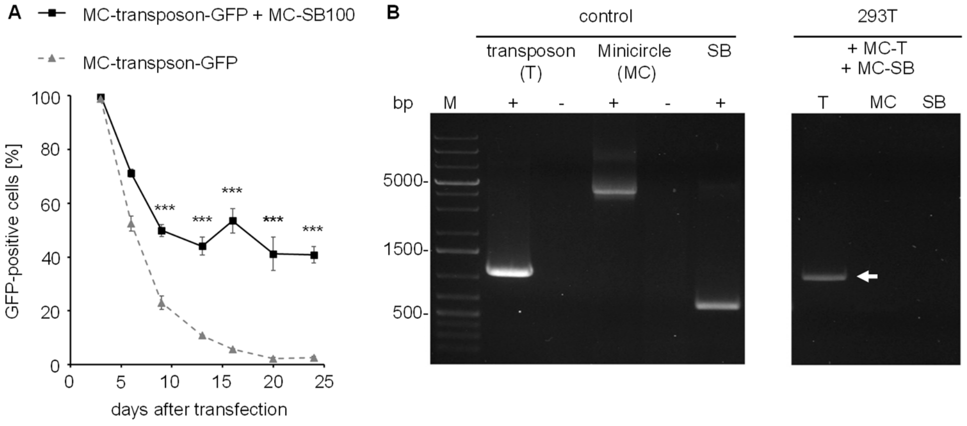

Figure S3 A: Characterization of the two-plasmid SB transposon system. 293T cells were either transfected simultaneously with MC-transposon-GFP and MC-SB100 or only with MC-transposon-GFP. The GFP expression was analyzed regularly via flow cytometry up to 25 days after transfection (n =3, mean ± SD). B: Cell lysates transfected with the two-plasmid SB transposon system were analyzed by PCR for expression of transposon (T), minicircle vector backbone (MC) and transposase SB100X (SB). As control DNA of T, MC and SB was used. Arrow depicts transposon-GFP integration into 293T.

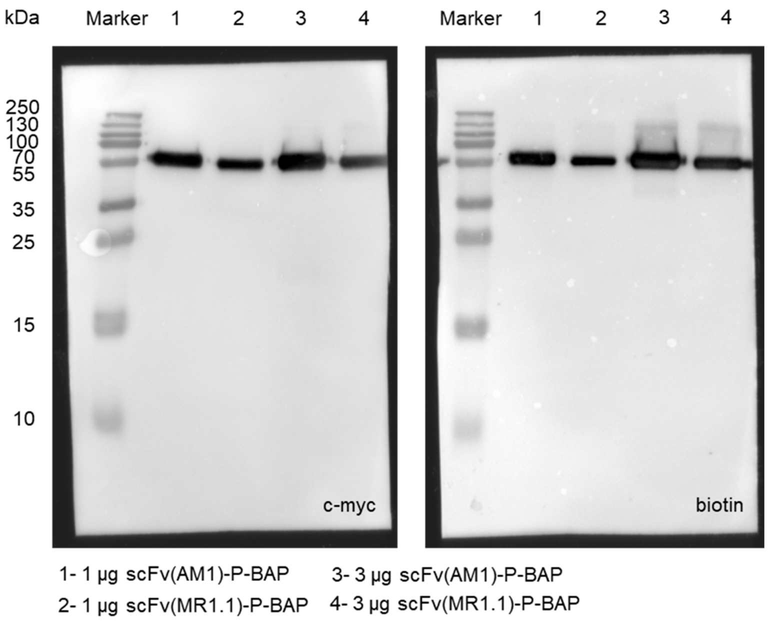

Figure S4 Original unedited representative image corresponding to the Western Blot presented in figure 2B.

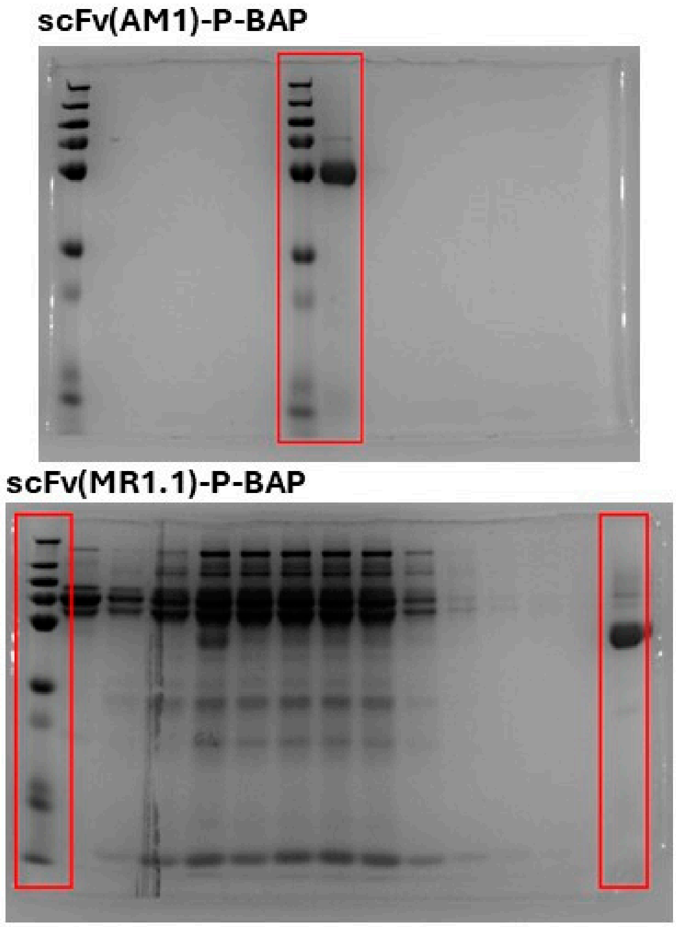

Figure S5 Original unedited representative image corresponding to the Coomassie-stained PAGE gels presented in figure 2C.

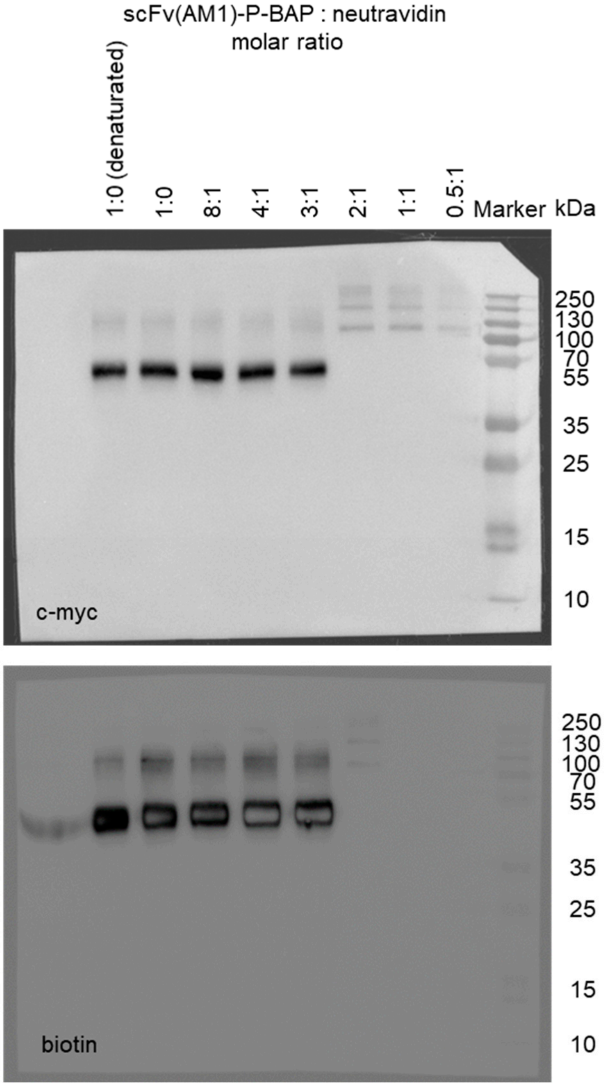

Figure S6 Original unedited representative image corresponding to the Western Blot presented in figure 3A.

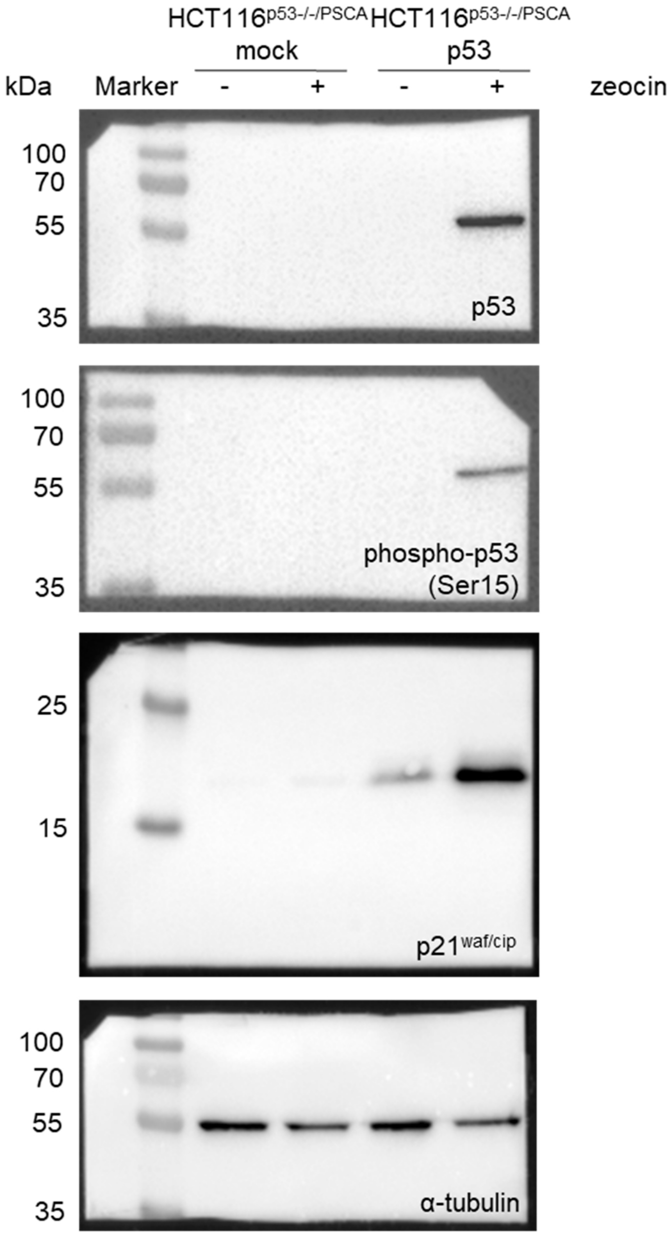

Figure S7 Original unedited representative image corresponding to the Western Blot presented in figure 5C.
